# Application of [18F]FLT‐PET in pulmonary arterial hypertension: a clinical study in pulmonary arterial hypertension patients and unaffected bone morphogenetic protein receptor type 2 mutation carriers

**DOI:** 10.1177/20458940211028017

**Published:** 2021-06-01

**Authors:** Liza Botros, Samara M.A. Jansen, Ali Ashek, Onno A. Spruijt, Jelco Tramper, Anton Vonk Noordegraaf, Jurjan Aman, Hans Harms, Frances S. de Man, Marc C. Huisman, Lan Zhao, Harm J. Bogaard

**Affiliations:** ^1^ Department of Pulmonology Amsterdam UMC Vrije Universiteit Amsterdam Amsterdam The Netherlands; ^2^ Faculty of Medicine National Heart and Lung Institute Imperial College London Hammersmith Hospital London UK; ^3^ Cardiovascular Imaging Program Departments of Radiology and Medicine; Division of Nuclear Medicine and Molecular Imaging Brigham and Women’s Hospital and Harvard Medical School Boston MA USA; ^4^ Institute of Clinical Medicine Aarhus University Hospital Aarhus Denmark; ^5^ Department of Radiology and Nuclear Medicine Amsterdam UMC Vrije Universiteit Amsterdam Amsterdam The Netherlands

**Keywords:** pulmonary hypertension, clinical diagnosis, pathogenesis, clinical outcome

## Abstract

Pulmonary arterial hypertension is a heterogeneous group of diseases characterized by vascular cell proliferation leading to pulmonary vascular remodelling and ultimately right heart failure. Previous data indicated that 3′‐deoxy‐3′‐[18F]‐fluorothymidine (^18^FLT) positron emission tomography (PET) scanning was increased in pulmonary arterial hypertension patients, hence providing a possible biomarker for pulmonary arterial hypertension as it reflects vascular cell hyperproliferation in the lung. This study sought to validate ^18^FLT‐PET in an expanded cohort of pulmonary arterial hypertension patients in comparison to matched healthy controls and unaffected bone morphogenetic protein receptor type 2 mutation carriers. ^18^FLT‐PET scanning was performed in 21 pulmonary arterial hypertension patients (15 hereditary pulmonary arterial hypertension and 6 idiopathic pulmonary arterial hypertension), 11 unaffected mutation carriers and 9 healthy control subjects. In‐depth kinetic analysis indicated that there were no differences in lung ^18^FLT k3 phosphorylation among pulmonary arterial hypertension patients, unaffected bone morphogenetic protein receptor type 2 mutation carriers and healthy controls. Lung ^18^FLT uptake did not correlate with haemodynamic or clinical parameters in pulmonary arterial hypertension patients. Sequential ^18^FLT‐PET scanning in three patients demonstrated uneven regional distribution in ^18^FLT uptake by 3D parametric mapping of the lung, although this did not follow the clinical course of the patient. We did not detect significantly increased lung ^18^FLT uptake in pulmonary arterial hypertension patients, nor in the unaffected bone morphogenetic protein receptor type 2 mutation carriers, as compared to healthy subjects. The conflicting results with our preliminary human ^18^FLT report may be explained by a small sample size previously and we observed large variation of lung ^18^FLT signals between patients, challenging the application of ^18^FLT‐PET as a biomarker in the pulmonary arterial hypertension clinic.

## Introduction

Pulmonary arterial hypertension (PAH) is a heterogeneous group of diseases that is characterized by obliteration and narrowing of the vascular lumen, leading to increased pulmonary vascular resistance (PVR) and ultimately right heart failure.^1^, ^2^ ^–^ ^3^ Featuring in the pathobiology of PAH are phenomena also encountered in cancer, such as enhanced proliferation, apoptosis‐resistance,^4^, ^5^ ^–^ ^6^ altered mitochondrial metabolism^7^ and chronic inflammation,^8^ although it is still unclear whether this is true in all PAH patients and at all stages of the disease. One of the most important risk factors for the development of PAH is a mutation in the gene coding for the bone morphogenetic protein receptor type 2 (BMPR2).^9^ Subjects carrying this pathogenic variant have an increased risk to develop PAH, but currently there are no methods to predict which unaffected mutation carriers will develop PAH. To date, screening and monitoring of disease progression rely on indicators of right ventricular dysfunction^10^ and no biomarkers of pulmonary vascular remodelling in PAH are available. Thus, there is an urgent need for non‐invasive quantification of vascular remodelling in patients with PAH or in subjects at risk.^11^

Recently, several studies used positron emission tomography (PET) to provide anatomical and metabolic information in the PAH lung.^7^ ^,^ ^12^, ^13^, ^14^ ^–^ ^15^ In animal models of PAH, uptake of fluorine‐18‐labelled 2‐fluoro‐2‐deoxyglucose FDG (^18^FDG), a marker of glucose metabolism, correlated with disease severity.^13^ Increased mean lung parenchymal uptake was also observed in a cohort of 20 patients with PAH (18 with idiopathic PAH (IPAH)).^13^ In another recent study, cardiac and lung standardized uptake value of ^18^FDG was found higher in 30 PH patients (9 IPAH, the rest of various PH groups) compared to healthy controls.^16^ As ^18^FDG measures glucose metabolism, it represents proliferative vascular cells as well as inflammatory cell accumulation, limiting its potential in measuring vascular remodelling in humans directly.^13^ ^,^ ^17^ It is recognized that ^18^FDG PET lacks specificity as a clinical tool for the diagnosis of PAH; furthermore, lung ^18^FDG uptake did not correlate with any clinical marker in PH patients.^13^ ^,^ ^16^ 3′‐deoxy‐3′‐[18F]‐fluorothymidine (^18^FLT), a thymidine analogue, has recently emerged as a more sensible tracer for imaging lung vascular proliferation in PAH.^12^ ^18^FLT is routinely used in clinical oncology^18^ as it depicts tumour growth in a variety of malignancies,^19^ and correlates with histological proliferation markers such as Ki‐67 and proliferating cell nuclear antigen.^20^ ^,^ ^21^ ^18^FLT phosphorylation by thymidine kinase 1 leads to ^18^FLT retention within the cell, thereby providing a quantitative measurement of proliferating tissue.^20^

We previously showed increased ^18^FLT uptake in pre‐clinical models of PAH and in preliminary clinical data with a small group (*n* = 8) of IPAH patients.^12^ We compared ^18^FLT uptake from the contralateral lung of patients with one‐sided pulmonary malignancies as control subjects. Herein, we conducted an expanded clinical ^18^FLT‐PET study (a) to include a larger group of PAH patients and an appropriate control group of healthy subjects, aiming to validate the clinical application of ^18^FLT‐PET as a biomarker for PAH severity and (b) to include a group of unaffected BMPR2 mutation carriers, aiming to assess if ^18^FLT‐PET can discriminate carriers that may be at risk of developing the subclinical pulmonary vascular remodelling.

## Methods

### Patient population

This study was conducted in accordance with the principles of the Declaration of Helsinki. Approval was obtained from the Medical Ethical Review Committee of the VU University Medical Centre (2017.334) and informed consent was obtained from all subjects. All subjects were recruited between 2015 and 2019 and all PAH patients were diagnosed according to current guidelines.^3^ Our expanded cohort consisted of eight PAH patients (five heritable PAH (HPAH) and three IPAH) from previous published study^12^ with 13 newly scanned PAH patients (10 HPAH and three IPAH). We also studied 11 unaffected BMPR2 mutation carriers and 9 matched healthy controls (siblings without a BMPR2 pathogenic variant). Three PAH patients were scanned twice during their clinical course. The most recent ^18^FLT‐PET scan was used in the expanded cohort.

### Right heart catheterizations and cardiac magnetic resonance imaging

All patients underwent right heart catheterization (RHC) and cardiac magnetic resonance (CMR) imaging within six weeks of the ^18^FLT‐PET scan as previously described.^22^ In short, a 7F balloon‐tipped flow‐directed triple‐lumen Swan‐Ganz catheter (Edwards Lifesciences LLC, Irvine, CA) was inserted in the pulmonary artery (PA) via the jugular vein under local anaesthesia and constant electrocardiography monitoring, and measurements were performed according to the guidelines.^3^ With RHC mean pulmonary artery pressure (mPAP), PVR, mean right atrial pressure, pulmonary arterial wedge pressure (PAWP) and cardiac output (CO) were measured. CO was measured using the thermodilution method. PVR was calculated as 80 × (mPAP – PAWP)/CO. CO was indexed to body surface area and shown as cardiac index. CMR imaging scans were performed on a Siemens 1.5T Sonato or Avanto scanner (Siemens Medical Solutions, Erlangen, Germany). Right ventricular end‐diastolic volume index (RVEDVI), right ventricular end‐systolic volume index (RVESVI) and right ventricular ejection fraction (RVEF) from CMR imaging were collected. The load on the right ventricle (RV) was calculated, i.e. arterial elastance (Ea) = mPAP/stroke volume. Stroke volume was measured by CMR. Acquisition and post processing was performed following our standard protocol as described previously.^22^

### FLT‐PET scanning

Dynamic 60‐min ^18^FLT‐PET scanning was performed after a fasting period of six hours following the scanning protocol that was previously described.^12^ All participants were positioned supine with lungs and the aortic branch in the field of view of the Philips Ingenuity TF PET/CT (Philips Healthcare, Best, Netherlands). A cannula was placed in the arm vein for injection of radioisotope. First, a low‐dose computed tomography (CT) scan was performed in all subjects to correct for photon attenuation, scatter and lung densities. ^18^FLT was synthesized as described previously.^23^ After a bolus injection of 370MBq of ^18^FLT in 5 ml saline at 0.8 ml/s, blood samples were drawn at 5, 10, 20, 30, 40 and 60 min to correct the image‐derived plasma input function for radiolabelled metabolites (^18^F‐glucuronide).^12^ Dynamic PET imaging was started at the time of ^18^FLT intravenous injection for one hour and emission data were reconstructed into a 36‐frame format (1 × 10, 8 × 5, 4 × 10, 3 × 20, 5 × 30, 5 × 60, 4 × 150, 4 × 300 and 2 × 600 s) using the three‐Dimensional Row‐Action Maximum Likelihood Algorithm, applying all appropriate corrections for dead time, decay, scatter, attenuation and normalization.

### Kinetic modelling and data analysis

Plasma kinetic modelling was performed using Inveon Research Workplace software (Siemens Healthcare Molecular Imaging) which was fitted with a MATLAB‐based in‐house kinetic analysis software package (CLICKFIT). Since the Philips Ingenuity TF PET/CT scanner and software was updated during the conduction of patient recruitment of our expanded cohort, the plasma kinetic model calculated via MATLAB 5.3 software, which was used in the preliminary results, was not compatible anymore and all data from the expanded cohort were reconstructed and analysed using CLICKFIT. Whole‐lung tissue time–activity curve (TAC) was calculated from lung PET images co‐registered with region of interest (ROI) drawn on lung CT images, covering the lung volume with clearly visible boundaries adjusted by CT density thresholding. The ROI was finalized with 1‐cm automated erosion from the edge to prevent partial volume effects. To obtain arterial input function, ROI was drawn on the PA at the level of the pulmonary trunk using early frames. Metabolite‐corrected PA blood TAC was used as input function and whole lung TAC was used as output function to fit in the software package CLICKFIT. Per voxel, the K3 rate derived from a reversible two‐compartment 4k model was considered as proliferation rate and used as total lung ^18^FLT uptake. Typically, we have generated k3 value of 25,000 voxels (2 mm^3^ per voxel) from each human lung. Voxels with k3 ≤ 0 or k3 ≥ 1 were considered as noise and excluded (< 1% of total voxels). For mid‐lung ^18^FLT uptake, a 2‐cm region at the level of the bifurcation of the PA was used for analysis. For the correction of lung density (air‐correction), a CT‐generated lung segmentation was generated and used following Patlaks reversible model formula: Air correction = (1 – Vb)/(1 – Vb – Vair).^24^ Further analysis by three‐dimensional (3D) parametric mapping of computed per‐voxel ^18^FLT uptake, such as Texture Analysis, Heterogeneity Index and Metabolic Tumour Volume measurement were developed to measure the heterogeneity in tracer uptake per voxel level in the lung.

### Statistical analysis

Data were described as either mean (± standard deviation) for continuous variables and as absolute numbers (%) for categorical variables. Between‐group differences were compared using one‐way ANOVA with Bonferroni post‐hoc correction. Differences between control groups were tested using the Student t‐tests or chi‐square tests, after visually checking for normal distributions. Non‐normal distributed variables were LOG‐transformed or tested using Wilcoxon signed rank test. Correlations between ^18^FLT uptake (k3) and clinical variables were determined by linear regression analyses. Throughout the analyses, a *p*‐value of <0.05 was considered statistically significant. All statistical analyses were performed with R (R Core Team 2019 R version 3.6.1) and GraphPad Prism for Windows version 8 (GraphPad Software, La Jolla, CA).

## Results

### Characteristics of the subjects

A total of 21 PAH patients, 11 unaffected BMPR2 mutation carriers and 9 controls were studied. The baseline characteristics of all subjects are summarized in Table 1. The female to male ratio was 2:1. The control group was comparable in age, BMI and gender. As expected, NYHA class, NT‐proBNP concentration and RV volumes differed between the groups (all *p*‐values <0.001). The patients who were scanned in our preliminary report were similar in clinical characteristics (supplementary table 1).

**Table 1:** Baseline characteristics and haemodynamics.

	Healthy controls*n* = 9	Unaffected mutation carrier *n* = 11	PAH*n* = 21	*p*‐Values
Age (yr)	47 ± 17	46 ± 18	45 ± 13	0.953
Male, *n* (%)	4 (44%)	4 (36%)	7 (33%)	0.846
NYHA functional class, I/II/III/IV (*n*)	9/0/0/0	11/0/0/0	4/12/5/0	**<0.001**
BMI, kg/m^2^	28.1 ± 5.7	24.6 ± 4.1	24.8 ± 3.7	0.134
NT‐proBNP, ng/ml	–	21 (13–35)	229 (58–667)	0.001
Hb, mmol	8.9 ± 0.9	9.1 ± 0.8	9.3 ± 1.4	0.725
Medical history				
Malignancy	0 (0%)	0 (0%)	0 (0%)	NA
Hypertension	2 (22%)	0 (0%)	2 (10%)	0.249
Diabetes mellitus	0 (0%)	0 (0%)	1 (5%)	0.614
VTE/pulmonary embolism	1 (11%)	0 (0%)	1 (5%)	0.517
Treatment				
Treatment naïve, *n* (%)	9 (100%)	11 (100%)	1 (5%)^a^	**<0.001**
Monotherapy (ERA or PDE5I), *n* (%)	–	–	1 (5%)	–
Dual combination therapy (ERA + PDE5I or PDE5i + prostacyclin* 0), *n* (%)	–	–	13 (62%)	–
Triple combination therapy (ERA + PDE5I + prostacyclin), *n* (%)	–	–	6 (29%)	–
Haemodynamic characteristics				–
Heart rate, beats/min	74 ± 10	72 ± 11	75 ± 12	0.769
mPAP, mmHg	–	15 (14–17)	50 (42–57)	**<0.001**
PAWP, mmHg	–	9 ± 2	10 ± 3	0.291
mRAP, mmHg	–	5 ± 2	8 ± 3	0.039
PVRI, dyn.s/cm^–5^/m^2^	–	47 (41–66)	362 (281–498)	**<0.001**
Cardiac index, l/min/m^2^	–	3.0 ± 0.5	2.7 ± 0.7	0.241
Cardiac magnetic resonance imaging				
RVEDVI, ml/m^2^	72 (58–81)	56 (54–69)	92 (75–111)^a^	**<0.001**
RVESVI, ml/m^2^	30 (26–35)	22 (20–26)	48 (41–70)^a^	**<0.001**
RVEF, %	58 ± 6	61 ± 5	44 ± 12^a^	**<0.001**
LVEF, %	66 ± 5	66 ± 7	62 ± 13	0.444

Note: Data are given as mean (SD), median (IQR) or percentages.

^a^Significant differences depicted in bold in post‐hoc analysis.

BMI: body mass index; Hb: haemoglobin; VTE: venous thromboembolism; ERA: endothelin receptor antagonist; PDE5I: phosphodiesterase type 5 inhibitor; mRAP: mean right atrial pressure; mPAP: mean pulmonary arterial pressure; PVRI: indexed pulmonary vascular resistance; PAWP: pulmonary arterial wedge pressure; RVEDVI: indexed right ventricular end‐diastolic volume; RVESVI: indexed right ventricular end‐systolic volume; RVEF: right ventricular ejection fraction; LVEF: left ventricular ejection fraction; PAH: pulmonary arterial hypertension.

### ^18^FLT k3 phosphorylation rate

**Fig. 1:**
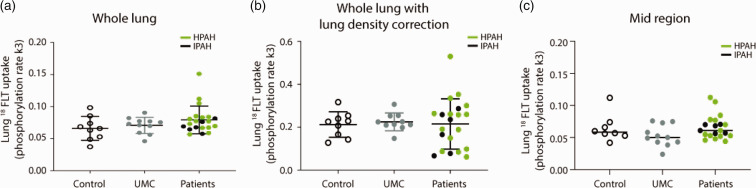
Lung ^18^FLT uptake was measured (phosphorylation rate k3 of ^18^FLT). No differences were observed between controls (*n* = 9), unaffected mutation carriers (UMC, *n* = 11) and PAH patients (*n* = 21) in whole lungs (a), whole lungs corrected for lung density measured with CT‐scan (b) and in the mid‐region of the lungs (c). ^18^FLT: 3′‐deoxy‐3′‐[18F]‐fluorothymidine; HPAH: heritable pulmonary arterial hypertension; IPAH: idiopathic pulmonary arterial hypertension.

For Kinetic modelling and data analysis, CLICKFIT was used. This method gave similar results in ^18^FLT‐uptake, expressed as k3 phosphorylation, in the PAH patients from the preliminary results for which MATLAB 5.3 software was used previously (supplemental Figure S1). Compared to lung ^18^FLT uptake in control subjects, the mean ^18^FLT phosphorylation k3 was not significantly different in PAH patients (Fig. 1a), so as in the unaffected BMPR2 mutation carriers. This unsignificant statistics of lung ^18^FLT uptake remained among the three groups after correction for lung density measured by CT (k3 score, Fig. 1b). We also analysed the ^18^FLT phosphorylation k3 in the mid‐region of the lungs^12^ and no significant differences were observed among the PAH patients, unaffected BMPR2 mutation carriers and controls (Fig. 1c). Stratification of the PAH patient groups considering age, subtype of PAH, NYHA class, therapy or co‐morbidity did not show correlation to lung ^18^FLT phosphorylation k3 measurements.

### Correlation with clinical parameters

**Fig. 2:**
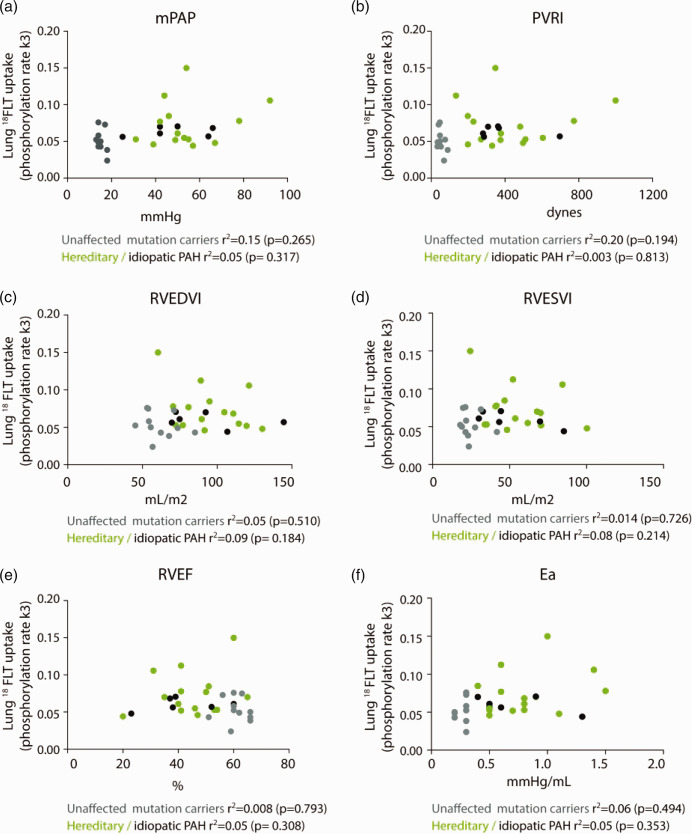
No correlation between ^18^FLT uptake (k3) in the mid‐region and mean pulmonary artery pressure (mPAP), indexed pulmonary vascular resistance (PVRI), right ventricular dimensions (RVEDVI and RVESVI), right ventricular function (RVEF) and load of the right ventricle (Ea). RVEDVI: indexed right ventricular end‐diastolic volume; RVESVI: indexed right ventricular end‐systolic volume; RVEF: right ventricular ejection fraction; Ea: arterial elastance; ^18^FLT: 3′‐deoxy‐3′‐[18F]‐fluorothymidine; PAH: pulmonary arterial hypertension.

We examined the relationship between the lung ^18^FLT uptake (k3) and cardiopulmonary haemodynamic parameters in PAH patients and unaffected BMPR2 mutation carriers. No correlation was observed between lung ^18^FLT uptake with mPAP and indexed PVR in PAH patients (*r* ^2^ = 0.05, *p* = 0.317 and *r* ^2^ = 0.003, *p* = 0.813 respectively) and in unaffected mutation carriers (*r* ^2^ = 0.15, *p* = 0.265 and *r* ^2^ = 0.20, *p* = 0.194 respectively). Lung ^18^FLT uptake also did not correlate with haemodynamic parameters by CMR, such as RVEDVI, RVESVI, RVEF and the load on the ventricle (arterial elastance; Fig. 2c to f).

### Follow‐up scans

**Fig. 3:**
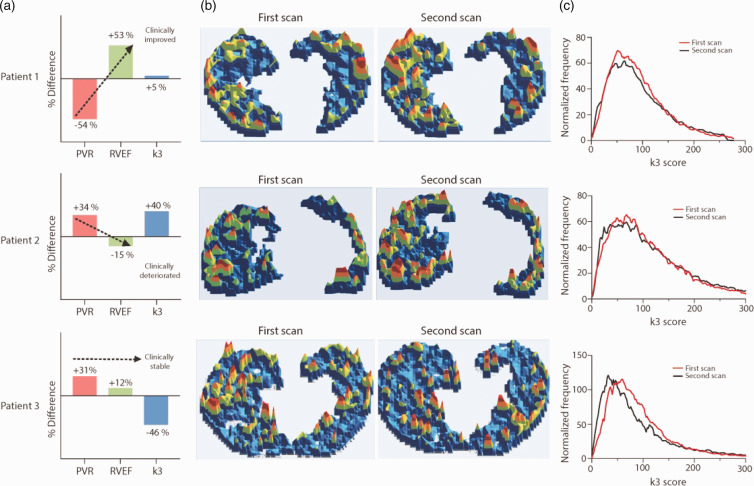
(a) % Difference in pulmonary vascular resistance (PVR), right ventricle ejection fraction (RVEF) and k3 phosphorylation rate (k3) in follow‐up scans from three PAH patients. (b) 3‐dimensional parametric map (axial view) generated from computed per‐voxel ^18^FLT lung uptake. Uneven regional ^18^FLT uptake was observed in all patients (blue colour = base line, green = low, yellow = mid and red = high). (c) Comparative histogram distribution of voxel wise FLT phosphorylation score for first and second scan. PVR: pulmonary vascular resistance; RVEF: right ventricular ejection fraction.

We performed further analysis by 3D parametric mapping in three PAH patients who were scanned sequentially (Fig. 3), aiming to define the previous observation of heterogeneous lung ^18^FLT uptake within the PAH group.^12^ Our data demonstrated the uneven regional ^18^FLT uptake (patch distribution) in the PAH patient lungs. Further analysis presented the comparative histogram distribution of voxel wise ^18^FLT phosphorylation rates of the two sequential ^18^FLT‐PET scans in the three PAH patients (Fig. 3). The comparative data indicated no significant differences between the two sequential scans in patients 1 and 2, but a left shift was observed in patient 3, indicating a reduced ^18^FLT phosphorylation rate in the second scan. The sequential lung^18^FLT uptake data did not relate to the haemodynamic parameters gained from these three PAH patients (Fig. 3 and Suppl. table 2).

## Discussion

This study conducted ^18^FLT‐PET in PAH patients in comparison with a group of healthy control subjects and unaffected BMPR2 mutation carriers. Kinetic modelling of the lung ^18^FLT uptake indicated that there were no significant differences of the lung ^18^FLT phosphorylation k3 in PAH patients comparing to the healthy control subjects. The lung ^18^FLT uptake in PAH patients did not correlate with the clinical and haemodynamical parameters. In depth 3D parametric mapping of the sequential ^18^FLT‐PET data from three PAH patients during their follow‐up showed uneven regional lung ^18^FLT uptake in these individuals. However, ^18^FLT lung uptake did not relate to the haemodynamic clinical parameter changes. We report for the first time that lung ^18^FLT uptake in unaffected BMPR2 mutation carriers, who are prone to develop PAH, are similar to the healthy control subjects.

The present study is a carefully designed follow‐up study of our previous ^18^FLT project, aiming to qualify the clinical application of ^18^FLT‐PET in the PH clinic. In the previous study, the control ^18^FLT lung uptake data were calculated from the contralateral healthy lung lobe of patients with one‐sided pulmonary malignancies. Three out of six cancer patients were receiving chemotherapy <1 month before ^18^FLT‐PET scanning. The current study employed an appropriate group of matched control subjects. These healthy subjects were younger than the previous control projects with cancer (age 47 vs 68 years, *p* = 0.015). Nevertheless, the present data demonstrated that the ^18^FLT lung uptake levels (k3) of these nine healthy control subjects are comparable to the previous demonstrated control ^18^FLT lung uptake levels. The current ^18^FLT‐PET study has expanded the previous small cohort study to a larger cohort of 21 PAH patients and produced an improved clinical data set. We demonstrated no significant differences in lung ^18^FLT uptake between the PAH patients and control healthy subjects, which conflicts with the preliminary human ^18^FLT‐PET study due to a small sample size previously and large variation of lung ^18^FLT signals between patients.

It remains a challenge to develop an appropriate PET tracer that detects, localizes and monitors pulmonary vascular pathology in PAH. ^18^FDG‐PET and ^18^FLT‐PET have been used in oncology to stratify patients and assess disease progression and response to treatment and predict clinical outcomes. Increased ^18^FDG lung signals with heterogeneous distribution were described in IPAH as well as in PAH patients due to systemic lupus erythematosus (SLE‐PAH),^13^ ^,^ ^14^ consistent with its property in tracking both proliferation and inflammation events. However, there were no significant correlations between lung ^18^FDG uptake with haemodynamic and severity parameters in IPAH patients detected,^14^ ^,^ ^15^ whilst data from SLE‐PAH patients confirmed that the increased lung ^18^FDG uptake reflects an active inflammatory disease status in the lung which impacts remodelling process. In comparison, ^18^FLT‐PET is recognized as a marker of evaluating whole tumour proliferation heterogeneity. We are challenged by the relatively lower ^18^FLT uptake compared to ^18^FDG^17^ and the large air volume in the lung, although ^18^FLT has a low background uptake in the thorax as shown in our previous data that indicated a relatively lower noise threshold than ^18^FDG.^25^ The present study applied the rate of ^18^FLT phosphorylation, k3, calculated from the in‐depth kinetic 2T4k modelling of the lung ^18^FLT signal for reflecting proliferation events in the lung^12^; however, k3 did not discriminate PAH patients from control healthy subjects, contradicting to our original hypothesis. Additionally, the unaffected BMPR2 mutation carriers presented a tighter mean of lung ^18^FLT phosphorylation k3 similar to the healthy control subjects.

We found that there is considerable larger variation in the distribution of lung ^18^FLT uptake in the PAH patients and there is visually heterogeneity in the lung ^18^FLT signal distribution within the lungs of each patient. Further stratification considering PH phenotype, therapy used, age, gender, duration of disease or haemodynamic parameters did not define any significant relevance with lung ^18^FLT phosphorylation k3. Comparing k3 phosphorylation of unaffected BMPR2 mutation carriers with healthy controls or PAH patients did not show differences. It remains difficult to state if FLT‐PET was unable to detect latent vascular proliferation, or if the lung vasculature of these mutation carriers is indeed unaffected until a second hit occurs. The data from sequential ^18^FLT‐PET scans that follows up three PAH patients under combination treatments (Prostacyclin, ERA, PDE5I) is informative; however, ^18^FLT lung uptake did not relate to haemodynamic or clinical parameter changes over time. Patient 3 started with prostacyclin treatment in between the sequential FLT‐PET scans, and his k3 value decreased impressively. It may be that a decrease in vessel wall remodelling was due to prostacyclin treatment,^26^ or that the vascular remodelling simply ‘burns out’ at a late phase of the disease. We are limited to make a direct correlation between ^18^FLT signal with pulmonary pathology in the same human study subjects; the natural history of the pulmonary pathology following clinical presentation is unknown. It may be episodic or particularly active at the start of the disease and reduced in the later phase. Evidence of vascular proliferation that is tracked by FLT imaging might be episodic or more evident in some patients than others irrespective of their clinical impairment at the time of imaging.

In the past decade, several clinical trials have targeted cancer‐like features in PAH. Inhibition of endothelial cell‐ and smooth muscle cell proliferation by for example tacrolimus^27^ and mercaptopurine^28^ showed some haemodynamic improvement, but in small patient groups and with serious side‐effects. As there is still no evidence of structurally abnormal growth factor receptors in the PAH vasculature, clinical trials with growth factor inhibitors such as tyrosine kinase inhibitors have not yet shown an overall clinical benefit.^29^, ^30^ ^–^ ^31^ This indicates that the general consensus of sustained proliferative signalling in PAH might be more nuanced and complex.

### Limitations

Several limitations of this study need to be recognized. A relatively small number of PAH patients and control subjects were included, which may affect the generalizability of the study. In addition, the majority of PAH patients received PAH‐specific therapy, mostly double therapy. As endothelin receptor blockage decreased pulmonary artery smooth muscle cell proliferation (PASMC) and improved microvessel density,^32^ and proliferation of PASMC was dose dependently reduced by both phosphodiesterase‐5 inhibitors^33^ and treprostinil,^26^ this may have mitigated small PA remodelling.

## Conclusion

The current study with a larger expanded PAH patient cohort established a valuable and critical clinical ^18^FLT‐PET data set. We could not detect significant differences in lung ^18^FLT phosphorylation rate k3 in PAH patients in comparison to the age‐matched healthy controls, nor in unaffected BMPR2 mutation carriers. Our data informed and challenged the application of ^18^FLT‐PET as a biomarker in PH clinic. Larger cohorts of PAH patients validating the use of ^18^FLT‐PET and novel tracers with a higher sensitivity for tracking PAH disease pathology are needed which may help to improve the detection of PAH and tailoring therapy strategies.

## Statement of author contributions

L.B., S.M.A.J., A.A., O.A.S., J.T., J.A., H.H., M.C.H., F.S.M., L.Z. and H.J.B. contributed to the conception and design of the study and interpretation of the data. L.B., S.M.A.J. and A.A. were responsible for data acquisition and analysis. L.B., S.M.A.J., A.A. and L.Z. drafted the manuscript. All authors critically revised the manuscript for important intellectual content and gave final approval of the version to be published. All authors are accountable for all aspects of the work.

## Ethical approval

Dutch medical ethical committee 2017.334. All participants signed informed consent.

## Conflict of Interest

The author(s) declare that there is no conflict of interest.

## Funding

This work was supported by the Netherlands Cardiovascular Research Initiative: an initiative with the support of the Dutch Heart Foundation (CVON2017‐4 DOLPHIN‐GENESIS). H.J.B., A.V.N. and F.S.M. received CVON grant (CVON2012‐08) from the Dutch Heart Foundation. L.Z. received funding from the British Heart foundation (PG/18/2/33446). The remaining authors have nothing to disclose.

## ORCID iD

Liza Botros https://orcid.org/0000‐0001‐8048‐0240

## Supporting information

Supplementary Material

Supplementary Material
