# Supplementary material for: Application of [18F]FLT‐PET in pulmonary arterial hypertension: a clinical study in pulmonary arterial hypertension patients and unaffected bone morphogenetic protein receptor type 2 mutation carriers
Source: Pulm Circ. 2021 Jun 1;11(3):20458940211028017. doi: 10.1177/20458940211028017 (PMC8256252; doi:10.1177/20458940211028017)
Supplement: Supplementary file 1 — Supplementary Material [file PUL2-11-20458940211028017-s002.pdf]

SUPPLEMENTAL MATERIAL

Supplemental table 1: Comparison between patients in the preliminary study and in the expanded dataset

	Preliminary study n=8	Expanded cohort n=13	p-value
Age (yr)	46 ± 12	44 ± 14	0.732
HPAH, n(%)	5 (63%)	9 (69%)	1.000
Male, n(%)	4 (50%)	3 (23%)	0.427
NYHA functional class, I/II/III/IV (n)	2/4/2/0	2/8/3/0	0.834
BMI, kg/m^2	24.6 ± 3.7	24.9 ± 3.7	0.825
NT-proBNP, ng/ml	190 [30-654]	229 [70-485]	0.943
**Medical history**			
Malignancy	0	0	NA
Hypertension	2 (25%)	0	0.259
Diabetes Mellitus	1 (13%)	0	0.802
VTE/pulmonary embolism	0	1 (8%)	1.000
**Treatment**			
Treatment Naïve, n(%)	1 (13%)	0	0.802
Monotherapy (ERA or PDE5I), n(%)	1 (13%)	0	0.802
Dual combination therapy (ERA + PDE5I or PDE5i + prostacyclin* 0), n(%)	3 (38%)	11 (85%)	0.081
Triple combination therapy (ERA + PDE5I + prostacyclin), n(%)	3 (38%)	2 (15%)	0.530
**Hemodynamic characteristics**			
Heart rate, beats/min	79 ± 8	73 ± 14	0.236
Mean pulmonary artery pressure, mmHg	45 [44-55]	53 [42-57]	0.704
Pulmonary arterial wedge pressure, mmHg	9 [7-10]	12 [10-13]	**0.017**
Mean right atrial pressure, mmHg	6 [3-8]	9 [7-10]	0.117
Pulmonary vascular resistance, dyn.s/cm^{-5}	318 [222-366]	367 [308-511]	0.296
Cardiac index, l/min/m^2	2.9 ± 0.7	2.7 ± 0.7	0.470
**Cardiac magnetic resonance imaging**			
RVEDVI, ml/m^2	91 [79-101]	92 [75-111]	0.999
RVESVI, ml/m^2	49 [44-63]	48 [34-70]	0.329
RVEF, %	39 ± 11	47 ± 12	0.104
LVEF, %	63 ± 8	60 ± 16	0.565
**^{18}FLT PET K3 phosphorylation**			
Mid-region	0.081 [0.067-0.107]	0.057 [0.053-0.068]	0.053
Whole lung	0.079 [0.065-0.095]	0.076 [0.067-0.083]	0.828

Data are depicted as mean(SD), median [IQR] or percentages, , BMI: body mass index, VTE: venous thromboembolism, ERA: endothelin receptor antagonist, PDE5I: phosphodiesterase type 5 inhibitor, mRAP: mean right atrial pressure, RVEDVI: Indexed right ventricular end-diastolic volume, RVESVI: Indexed right ventricular end-systolic volume, RVEF: right ventricular ejection fraction, LVEF: left ventricular ejection fraction

Supplemental table 2: follow-up ^{18}FLT-PET scans in three pulmonary arterial hypertension patient

	Baseline characteristics			^{18}FLT-PET 2015 first scan							^{18}FLT-PET 2018 second scan							
	Age	Gender	BMI	mPAP	PVR	RVEDVI	RVESVI	RVEF	k3 mid-region	Medication	mPAP	PVR	RVEDVI	RVESVI	RVEF	k3 mid-region	Change in medication	12-months follow up
Patient 1	54	female	24.5	25	489	69	43	38	0.056	Prostacyclin + PDE5I	26	176	65	27	58	0.059	+ ERA	deteriorated mildly
Patient 2	68	female	18.3	44	548	94	55	41	0.050	ERA + PDE5I	50	732	105	68	35	0.070	none	Severe deterioration and died after stroke
Patient 3	43	male	24.4	69	762	126	105	17	0.150	ERA + PDE5I	67	998	130	100	19	0.081	+ prostacyclin	Severe deterioration, received lung transplantation and died after transplantation

mPAP: mean pulmonary artery pressure, PVR: pulmonary vascular resistance, RVESVI: Indexed right ventricular end-systolic volume; RVEDVI: Indexed right ventricular end-diastolic volume, RVEF: right ventricular ejection fraction, ERA: endothelin receptor antagonist, PDE5I: phosphodiesterase type 5 inhibitor
