# Supplementary material for: Application of [18F]FLT‐PET in pulmonary arterial hypertension: a clinical study in pulmonary arterial hypertension patients and unaffected bone morphogenetic protein receptor type 2 mutation carriers
Source: Pulm Circ. 2021 Jun 1;11(3):20458940211028017. doi: 10.1177/20458940211028017 (PMC8256252; doi:10.1177/20458940211028017)
Supplement: Supplementary file 2 — Supplementary Material [file PUL2-11-20458940211028017-s001.pdf]

Supplemental Figure 1

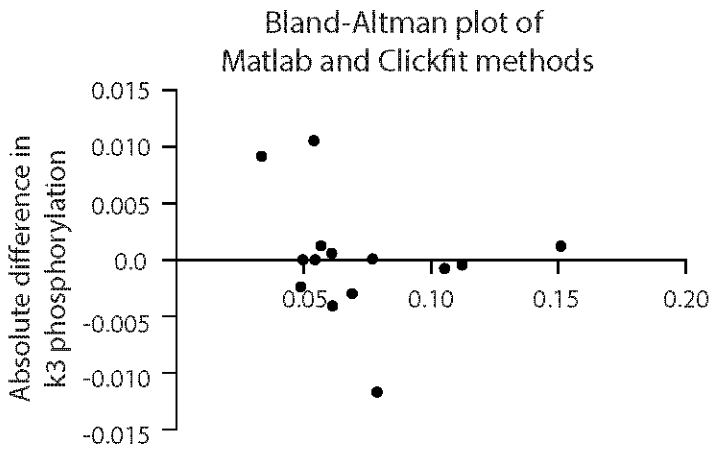

Supplemental Figure 1: Bland-Altman plot comparing two differend methods to calculate FLT-uptake in the lung.
In PAH patients from the preliminary cohort, kinetic modelling using CLICKFIT gave similar results as the MATLAB 5.3 software.
